# Comparison of Phototherapy and Exchange Transfusion Thresholds According to the Turkish Neonatology Society and the 2022 American Academy of Pediatrics Guidelines in Neonates with Indirect Hyperbilirubinemia

**DOI:** 10.3390/children13040540

**Published:** 2026-04-13

**Authors:** Deniz Keskindil, Senem Alkan Ozdemir, Şebnem Çalkavur, Tülin Gökmen Yildirim

**Affiliations:** HSU İzmir Dr. Behçet Uz Children’s Diseases and Surgery Training and Research Hospital, Konak 35210, Turkey; denizkeskindil.2045@outlook.com (D.K.); sebnemcalkavur@yahoo.com (Ş.Ç.); drtuling@yahoo.com.tr (T.G.Y.)

**Keywords:** indirect hyperbilirubinemia, guideline, neonatal

## Abstract

**Highlights:**

**What are the main findings?**

- Phototherapy and exchange transfusion thresholds were significantly higher when calculated according to the 2022 American Academy of Pediatrics guideline compared with the Turkish Neonatology Society guideline.
- A substantial proportion of neonates hospitalized for indirect hyperbilirubinemia met admission criteria according to the national guideline but not according to the 2022 American Academy of Pediatrics guideline.

**What are the implications of the main findings?**

- Differences between national and international guidelines may lead to considerable variation in hospitalization practices in neonatal intensive care units.
- Real-life comparative data may support future evaluations of guideline-based management strategies for neonatal hyperbilirubinemia.

**Abstract:**

**Background:** Clinical management of neonatal indirect hyperbilirubinemia is guided by threshold-based recommendations for phototherapy and exchange transfusion. This retrospective, single-center study compared phototherapy and exchange transfusion thresholds according to the Turkish Neonatology Society and the 2022 American Academy of Pediatrics guidelines in neonates hospitalized for indirect hyperbilirubinemia. **Methods:** This single-center, retrospective cross-sectional study included neonates born at ≥35 weeks of gestation who were admitted to a neonatal intensive care unit solely due to indirect hyperbilirubinemia. Phototherapy and exchange transfusion thresholds were calculated according to both the TNS guideline and the 2022 AAP guideline. Eligibility according to guideline thresholds and admission indications were compared between the two guidelines. Statistical analyses were performed using appropriate non-parametric tests. **Results:** A total of 344 neonates were included in the analysis. Mean phototherapy and exchange transfusion thresholds were significantly higher according to the AAP 2022 guideline compared with the TNS guideline (*p* < 0.001 for both). While 89.2% of admissions met eligibility according to the national guideline thresholds, only 36.6% met admission criteria according to the AAP 2022 guideline. **Conclusions:** Substantial differences exist between national and international guidelines for the management of neonatal indirect hyperbilirubinemia. These differences significantly influence treatment thresholds and hospitalization practices. Real-life comparative data may contribute to future evaluations of guideline-based management strategies.

## 1. Introduction

Neonatal jaundice remains one of the most common causes of hospital admission during the early postnatal period. Although the majority of cases are benign and self-limited, severe indirect hyperbilirubinemia may lead to bilirubin-induced neurological dysfunction (BIND) and kernicterus if not recognized and treated appropriately [1]. Therefore, timely identification and management of hyperbilirubinemia continue to be a critical component of neonatal care. Clinical decision-making in neonatal hyperbilirubinemia largely depends on guideline-defined thresholds for phototherapy and exchange transfusion. These thresholds aim to balance the prevention of bilirubin neurotoxicity with the potential risks of overtreatment [1,2]. Consequently, updates to international guidelines have significant implications for daily clinical practice.

In 2022, the American Academy of Pediatrics (AAP) updated its clinical practice guideline for the management of hyperbilirubinemia in infants born at ≥35 weeks of gestation [3]. Compared with previous recommendations, the updated guideline raised both phototherapy and exchange transfusion thresholds, reflecting accumulating evidence that higher bilirubin levels may be tolerated safely in selected neonates without increasing the risk of neurotoxicity [3,4]. In addition, the AAP emphasized individualized risk assessment, incorporating gestational age and specific neurotoxicity risk factors into treatment decisions.

In Türkiye, neonatal hyperbilirubinemia is managed according to the national guideline published by the Turkish Neonatology Society (TNS) [5]. While this guideline has been widely adopted in clinical practice, it differs from the updated AAP recommendations, particularly regarding treatment thresholds for phototherapy and exchange transfusion. These differences may lead to variation in admission practices and treatment decisions, especially in neonatal intensive care units (NICUs).

However, real-life data directly comparing the practical implications of these guidelines remain limited. In particular, there is a lack of studies assessing how differences in treatment thresholds translate into hospital admission rates among neonates with indirect hyperbilirubinemia [6].

The aim of this study was to compare phototherapy and exchange transfusion thresholds according to the TNS guideline and the 2022 AAP guideline in neonates born at ≥35 weeks of gestation who were admitted to a NICU with indirect hyperbilirubinemia. By evaluating eligibility according to guideline thresholds and potential differences in admission indications, we sought to highlight the clinical implications of applying national versus updated international recommendations in routine neonatal care.

## 2. Materials and Methods

### 2.1. Study Design and Setting

This study was designed as a single-center, retrospective cross-sectional analysis. This study was conducted in the Neonatal Intensive Care Unit (NICU) of HSU İzmir Dr. Behçet Uz Children’s Diseases and Surgery Training and Research Hospital, a tertiary referral center. Medical records of neonates hospitalized with a diagnosis of indirect hyperbilirubinemia were reviewed.

### 2.2. Study Population

Neonates admitted to the NICU during the study period were identified using relevant ICD-10 diagnostic codes. Our institution is a tertiary pediatric referral hospital without maternity services; therefore, all neonates included in this study were born in external maternity hospitals and referred to our center due to indirect hyperbilirubinemia. Infants born at ≥35 weeks of gestation who were hospitalized solely due to indirect hyperbilirubinemia were eligible for inclusion. Eligibility according to guideline thresholds was evaluated retrospectively for each neonate by jointly considering gestational age, postnatal age at admission, total serum bilirubin level, and documented neurotoxicity risk factors. These variables were compared individually with both the Turkish Neonatology Society and the 2022 American Academy of Pediatrics guideline thresholds to determine theoretical eligibility for admission and treatment. Thresholds were calculated at the time of admission for each individual neonate.

Inclusion criteria were:

- Gestational age ≥ 35 weeks;
- Admission to the NICU due to indirect hyperbilirubinemia;
- Absence of major comorbid conditions.

Exclusion criteria included:

- Presence of additional systemic diseases;
- Incomplete medical records;
- Admission for reasons other than indirect hyperbilirubinemia.

After applying inclusion and exclusion criteria, a total of 344 neonates were included in the final analysis.

### 2.3. Data Collection

Demographic, clinical, and laboratory data were obtained from electronic medical records and patient files. The following variables were recorded: gestational age, birth weight, sex, postnatal age at admission, feeding type, duration of hospitalization, and duration of phototherapy.

Laboratory parameters included total serum bilirubin (TSB) levels at admission, direct Coombs test results, blood group incompatibilities (ABO and Rh), reticulocyte count, C-reactive protein (CRP), thyroid function tests (TSH and free T4), glucose-6-phosphate dehydrogenase (G6PD) deficiency status, blood culture results, and presence of urinary tract infection.

### 2.4. Guideline-Based Assessment

Phototherapy and exchange transfusion thresholds were determined according to both guidelines. Guideline-based admission, phototherapy, and exchange transfusion thresholds were applied retrospectively to each neonate using recorded clinical and laboratory data. These assessments reflected theoretical eligibility according to guideline criteria and were performed independently of the actual clinical decisions made at the time of hospitalization. The retrospective application was intended to allow a standardized comparison between guidelines rather than to evaluate real-time clinical judgment.

Eligibility according to guideline thresholds was evaluated by determining whether the measured TSB level at admission met the respective phototherapy or exchange transfusion thresholds. Guideline-based admission indication rates were compared between the two recommendations.

### 2.5. Outcomes

The primary outcome was the difference in phototherapy and exchange transfusion thresholds between the TNS and AAP 2022 guidelines. Secondary outcomes included eligibility according to guideline thresholds rates and potential differences in admission indications according to gestational age.

While subgroup analyses by gestational age may further enhance data visualization, the present study focused on overall guideline-based eligibility to maintain consistency with its primary objective.

### 2.6. Statistical Analysis

Statistical analyses were performed using SPSS version 25.0 (IBM Corp., Armonk, NY, USA). Continuous variables were assessed for normal distribution using the Shapiro–Wilk test. Data were presented as mean ± standard deviation or median (interquartile range), as appropriate. Categorical variables were expressed as numbers and percentages. Paired comparisons of phototherapy and exchange transfusion threshold values between the two guidelines were conducted using the Wilcoxon signed-rank test. Comparisons of hospitalization and phototherapy durations across gestational age groups were performed using the Kruskal–Wallis test. A *p*-value < 0.05 was considered statistically significant.

## 3. Results

A total of 510 neonates admitted to the neonatal intensive care unit during the study period were initially screened. Of these, 100 neonates were excluded due to unavailable medical records and 66 neonates did not meet the inclusion criteria. Consequently, a total of 344 neonates born at ≥35 weeks of gestation and hospitalized solely due to indirect hyperbilirubinemia were included in the final analysis.

### 3.1. Baseline Characteristics

Baseline demographic and clinical characteristics of the study population are summarized in Table 1. The mean gestational age of the included neonates was 37.7 ± 1.43 weeks, and the mean birth weight was 3234.6 ± 1372.5 g. Of the infants, 54.4% were male. The median postnatal age at admission for jaundice was 5 days. The mean total serum bilirubin level at initiation of treatment was 17.88 ± 4.92 mg/dL. The mean duration of hospitalization was 4.46 ± 3.21 days, and the mean duration of phototherapy was 17.52 ± 10.87 h (Table 1).

### 3.2. Clinical and Laboratory Findings

Categorical clinical and laboratory characteristics are presented in Table 2. ABO incompatibility was present in 21.5% of the neonates, while Rh incompatibility was observed in 6.1%. Direct Coombs test positivity was detected in 7.3% of cases. Glucose-6-phosphate dehydrogenase (G6PD) deficiency was identified in 9.7% of the neonates. Blood culture positivity was detected in 2.6% of cases; however, these results were interpreted as contamination. Urinary tract infection was identified in 13.4% of the infants. Exchange transfusion was performed in 2.0% of the study population. The majority of neonates (89.0%) were exclusively breastfed (Table 2).

### 3.3. Comparison of Guideline Thresholds

Phototherapy and exchange transfusion threshold values according to the Turkish Neonatology Society (TNS) and the American Academy of Pediatrics (AAP) 2022 guidelines are shown in Table 3. The mean phototherapy threshold according to the TNS guideline was 15.83 ± 2.46 mg/dL, whereas the mean phototherapy threshold according to the AAP 2022 guideline was 19.61 ± 2.31 mg/dL. This difference was statistically significant (*p* < 0.001). Similarly, the mean exchange transfusion threshold was 20.43 ± 2.43 mg/dL according to the TNS guideline and 25.76 ± 1.57 mg/dL according to the AAP 2022 guideline, this difference reached statistical significance (*p* < 0.001) (Table 3). Mean threshold values represent the average of guideline-specific phototherapy or exchange transfusion thresholds individually calculated for each neonate at the time of admission, rather than a single threshold applied uniformly across the cohort and presented in Table 3.

### 3.4. Eligibility According to Guideline Thresholds

Among the 344 neonates admitted due to indirect hyperbilirubinemia, 307 infants (89.2%) met the admission criteria according to the TNS guideline. In contrast, only 126 infants (36.6%) met the admission criteria based on the AAP 2022 guideline. When eligibility according to guideline thresholds was further evaluated according to gestational age, approximately 64.4% of the hospitalized neonates did not have an indication for admission according to the AAP 2022 guideline.

The distribution of major risk factors associated with bilirubin neurotoxicity is summarized in Table 4.

## 4. Discussion

The present study compared phototherapy and exchange transfusion thresholds defined by the Turkish Neonatology Society and the 2022 American Academy of Pediatrics guidelines in neonates hospitalized for indirect hyperbilirubinemia. The main finding of this study is the substantial difference between the two guidelines in terms of treatment thresholds and admission indications. Although stratification by gestational age, etiology, or risk profile may further enhance interpretability, the present analysis focused on overall guideline-based theoretical eligibility to maintain alignment with the study’s primary objective. Our results demonstrate that both phototherapy and exchange transfusion thresholds were significantly higher when calculated according to the AAP 2022 guideline compared with the TNS guideline. This finding is consistent across the entire study population and reflects the more conservative approach of the national guideline in comparison with the updated international recommendations [1,2,7]. One of the most striking observations in our study is the marked difference in eligibility according to guideline thresholds rates. While the majority of neonates admitted with indirect hyperbilirubinemia met the admission criteria according to the TNS guideline, less than half of these admissions met eligibility according to the AAP 2022 guideline. Furthermore, when evaluated according to gestational age, approximately two-thirds of the hospitalized neonates did not meet the admission criteria based on the AAP 2022 recommendations. This discrepancy suggests that the use of different guidelines may significantly influence hospitalization practices in neonatal intensive care units [1,2,8]. Although statistically significant differences in treatment thresholds were observed, these differences primarily reflect the inherent design and philosophy of the respective guidelines rather than a direct measure of clinical superiority. Therefore, the clinical relevance of these findings lies in their potential impact on admission practices and care pathways, rather than in statistical significance alone.

National guidelines for the management of neonatal hyperbilirubinemia have traditionally adopted a more conservative approach, largely driven by the desire to minimize the risk of bilirubin-induced neurological dysfunction and kernicterus [9]. Given the potentially irreversible consequences of severe hyperbilirubinemia, earlier initiation of treatment and lower intervention thresholds may be favored to maximize patient safety [10]. This cautious strategy, while prioritizing neuroprotection, may also lead to increased rates of phototherapy initiation and NICU admissions, particularly in settings where close outpatient follow-up may be challenging [11,12]. Some countries continue to follow the NICE guideline, which adopts lower treatment thresholds but does not explicitly incorporate neurotoxicity risk stratification [13].

In contrast, the 2022 AAP guideline emphasizes an individualized, risk-based approach by incorporating gestational age and specific neurotoxicity risk factors into treatment decisions. It should be emphasized that the 2022 AAP guideline promotes individualized risk assessment and close follow-up, and meeting treatment thresholds does not necessarily mandate immediate hospital admission in all cases. Therefore, our findings should be interpreted as a comparison of guideline-based theoretical eligibility rather than a critique of clinical decision-making. Following the publication of the 2022 AAP guideline, several studies have examined real-world implementation in clinical settings [14]. Reported findings generally suggest reductions in serum bilirubin testing and phototherapy utilization after adoption of the updated thresholds, reflecting a shift toward more selective treatment [15]. In addition, large database and hospital-based analyses have reported decreased hospitalization for jaundice in the period following the guideline update, while emphasizing the need for continued surveillance of safety outcomes [16]. Quality-improvement and rapid-implementation reports similarly describe practice changes aligned with the updated recommendations, often focusing on reducing subthreshold phototherapy and optimizing inpatient versus outpatient management pathways [17].

In this context, our study contributes complementary evidence by quantifying how applying AAP 2022 versus national guideline thresholds would change theoretical eligibility for admission and treatment in a pediatric referral hospital population. Importantly, our findings should be interpreted as a comparison of guideline frameworks and admission criteria rather than outcome-based evidence to support changing practice, highlighting the need for prospective studies incorporating standardized follow-up and clinical outcomes. The upward revision of treatment thresholds reflects growing evidence that higher bilirubin levels can be safely tolerated in selected neonates when appropriate monitoring and follow-up are ensured [18]. Importantly, available data indicate that the implementation of higher thresholds has not been associated with an increased incidence of bilirubin-related neurological complications, supporting the safety of this approach. The observed differences between the two guidelines may also have implications for healthcare resource utilization. Admission to a neonatal intensive care unit is associated with increased healthcare costs, longer hospital stays, and separation of the newborn from the family [19,20,21]. In this context, our finding that a substantial proportion of admitted neonates did not meet AAP 2022 admission criteria suggests that guideline selection may influence not only clinical management but also NICU workload and family-centered outcomes. Nevertheless, patient safety remains the primary concern, and any reduction in hospitalization should be accompanied by reliable systems for early detection, monitoring, and follow-up. Although risk factors for bilirubin neurotoxicity were documented, the present study was not designed to perform stratified analyses based on individual high-risk categories. Future multicenter studies incorporating risk-stratified analyses may further clarify how guideline-based thresholds interact with specific neurotoxicity risk profiles. Despite the absence of outcome data, the present findings provide insight into how differing guideline thresholds may influence initial hospitalization practices in pediatric referral centers.

From a public health perspective, differences in guideline-based phototherapy and exchange transfusion thresholds may have important implications for healthcare resource utilization and neonatal care pathways. In pediatric referral centers, lower treatment thresholds may lead to increased hospitalization rates, higher neonatal intensive care unit occupancy, and greater separation of newborns from their families. Real-life comparative data, such as those presented in this study, may contribute to preventive strategies by supporting risk-adapted admission policies, promoting appropriate use of hospital resources, and informing follow-up-based management approaches for neonatal hyperbilirubinemia. These considerations are particularly relevant for healthcare systems aiming to balance patient safety with efficient and family-centered care.

This study has several strengths, including a relatively large sample size and the use of real-life clinical data from a tertiary neonatal intensive care unit. However, certain limitations should be acknowledged. The retrospective and single-center design may limit the generalizability of the findings. In addition, long-term neurological outcomes were not assessed, which precludes direct evaluation of neurodevelopmental safety associated with different treatment thresholds. Post-discharge follow-up and readmission outcomes were not evaluated in this study and should be addressed in future studies focusing on the continuum of care in neonatal hyperbilirubinemia [22]. The present study focused on guideline-based theoretical eligibility for hospitalization and treatment and did not evaluate clinical outcomes such as readmission rates, rebound bilirubin levels, or long-term neurologic follow-up. Owing to the retrospective design and referral-based patient population, standardized post-discharge outcome data were not uniformly available. These outcome measures represent an important area for future prospective studies assessing the real-world impact of guideline-based management strategies.

## 5. Conclusions

This study provides real-life comparative data on phototherapy and exchange transfusion thresholds according to the Turkish Neonatology Society and the 2022 American Academy of Pediatrics guidelines in neonates hospitalized for indirect hyperbilirubinemia. Substantial differences were observed between the two guidelines, with higher treatment thresholds and fewer admission indications when applying the updated international recommendations. Importantly, these findings should not be interpreted as evidence to support changes in clinical practice in the absence of outcome-based data, but rather as real-life observations highlighting how differing guideline frameworks may shape hospitalization decisions in pediatric referral centers.

Our findings suggest that guideline selection may significantly influence hospitalization practices in pediatric referral centers managing neonatal hyperbilirubinemia. These results highlight the importance of critically evaluating guideline-based admission criteria in real-world settings and underscore the need for further multicenter studies incorporating post-discharge follow-up to optimize neonatal jaundice management.

## Figures and Tables

**Table 1:** Baseline demographic and clinical characteristics of the study population.

Variable	n	Mean	SD	25th Percentile	Median	75th Percentile
Gestational age (weeks) *	344	37.74	1.43	37.00	38.00	39.00
Birth weight (g) *	344	3234.6	1372.5	2826.3	3161.0	3468.8
Postnatal age at admission (days) *	344	6.30	5.08	3.00	5.00	7.00
TSH (mIU/L) *	321	4.99	4.29	2.26	3.75	6.37
Free T4 (ng/dL) *	324	1.62	0.43	1.31	1.56	1.91
CRP (mg/dL) *	344	0.16	0.34	0.02	0.06	0.13
Total serum bilirubin at treatment initiation (mg/dL) *	344	17.88	4.92	15.60	17.50	19.57
Length of hospital stay (days) *	344	4.46	3.21	2.00	3.00	5.00
Duration of phototherapy (hours) *	344	17.52	10.87	10.00	15.00	24.00
Reticulocyte count (%) *	342	2.80	2.40	1.13	2.00	3.79

* Continuous variables are presented as mean ± standard deviation and percentiles.

**Table 2:** Categorical clinical and laboratory characteristics of the study population.

Variable	Category	n	%
Sex *	Male	187	54.4
	Female	157	45.6
ABO incompatibility *	Absent	270	78.5
	Present	74	21.5
Rh incompatibility *	Absent	323	93.9
	Present	21	6.1
Direct Coombs test *	Negative	319	92.7
	Positive	25	7.3
Blood culture positivity *	Absent	335	97.4
	Present	9	2.6
G6PD deficiency *	Absent	307	90.3
	Present	33	9.7
Urinary tract infection *	Absent	297	86.6
	Present	46	13.4
Exchange transfusion *	Not performed	337	98.0
	Performed	7	2.0
Breastfeeding *	No	38	11.0
	Yes	306	89.0
Formula feeding *	No	306	89.0
	Yes	38	11.0

* Categorical variables are presented as number (percentage).

**Table 3:** Comparison of phototherapy and exchange transfusion thresholds according to the Turkish Neonatology Society and AAP 2022 guidelines.

Variable	n	Mean	SD	25th Percentile	Median	75th Percentile	*p*-Value *
TNS phototherapy threshold (mg/dL) *	344	15.83	2.46	15.00	15.70	18.00	**<0.001**
AAP 2022 phototherapy threshold (mg/dL) *	344	19.61	2.31	19.00	20.00	21.00	
TNS exchange transfusion threshold (mg/dL) *	344	20.43	2.43	19.00	21.00	22.50	**<0.001**
AAP 2022 exchange transfusion threshold (mg/dL) *	344	25.76	1.57	25.50	26.00	27.00	

* Paired comparisons between guideline thresholds were performed using the Wilcoxon signed-rank test.

**Table 4:** Distribution of major neurotoxicity risk factors in the study cohort.

Risk Factor	n (%)
Prematurity (<38 weeks)	35 (10.1)
ABO incompatibility	74 (21.5)
Rh incompatibility	21 (6.1)
Positive direct Coombs test	25 (7.3)
G6PD deficiency	33 (9.7)
Suspected or confirmed infection	55 (15.9)
Exclusive breastfeeding	306 (89)

## Data Availability

The data supporting the findings of this study are not publicly available due to ethical and privacy considerations related to patient data. However, anonymized datasets may be made available by the corresponding author upon reasonable request and subject to institutional approval.
